# Effectiveness of Indocyanine Green Fluorescence Angiography in Preventing Anastomotic Leakage: A Systematic Review and Meta-Analysis of Randomised Controlled Trials

**DOI:** 10.7759/cureus.94979

**Published:** 2025-10-20

**Authors:** Nirmani Widanage, Zain Ahmed Khan, Kasun Gunathilaka

**Affiliations:** 1 General and Colorectal Surgery, George Eliot Hospital NHS Trust, Nuneaton, GBR; 2 General Surgery, Billroth Hospitals, Chennai, IND; 3 Surgical Oncology, National Cancer Institute Sri Lanka, Maharagama, LKA

**Keywords:** anastomotic leakage, colectomy, colorectal surgery, fluorescence angiography, indocyanine green, intraoperative imaging, ischaemia, left-sided resection, perfusion assessment

## Abstract

Anastomotic leakage (AL) is one of the most serious complications after colorectal surgery, contributing to increased postoperative illness, extended hospitalisation, and higher mortality. Intra-operative evaluation of bowel perfusion with indocyanine green fluorescence angiography (ICG-FA) has emerged as a promising technique to reduce this risk, but randomised evidence remains limited.

A systematic review and meta-analysis was conducted according to Preferred Reporting Items for Systematic Reviews and Meta-Analyses (PRISMA) 2020 guidelines. PubMed, Excerpta Medica database (Embase) via Ovid, Medical Literature Analysis and Retrieval System Online (MEDLINE) via Ovid, ScienceDirect, and ClinicalTrials.gov databases were searched for randomised controlled trials (RCTs) comparing ICG-FA to conventional visual assessment during elective colorectal resections with primary anastomosis. The primary outcome assessed was AL. Subgroup analyses were performed for left- and right-sided resections. Risk of bias was assessed using the Cochrane Risk of Bias 2.0 (RoB 2) tool. A meta-analysis was conducted using a random-effects model, and heterogeneity was assessed using the I² statistic. Certainty of evidence was assessed using the Grading of Recommendations Assessment, Development and Evaluation (GRADE) approach.

Meta-analysis of seven RCTs involving 3,887 patients showed that ICG-FA significantly reduced anastomotic leak rates compared to standard visual assessment. The pooled odds ratio (OR) was 0.66 (95% CI: 0.53-0.82; p = 0.003), with no heterogeneity (I² = 0%). The estimated number needed to treat (NNT) to prevent one leak was 22. Subgroup analysis revealed a greater effect in left-sided resections (OR: 0.59; 95% CI: 0.46-0.75; p = 0.002; NNT = 18), with no significant benefit seen in right-sided cases (OR: 0.83; p = 0.35).

ICG-FA reduces anastomotic leak risk in colorectal surgery, particularly in left-sided resections. Further high-quality RCTs are needed to strengthen current evidence.

## Introduction and background

Anastomotic leakage (AL) remains a major postoperative challenge after colorectal surgery, with contemporary studies reporting rates of approximately 3%-19% [1]. This contributes to considerable postoperative morbidity and mortality, frequently resulting in re-operations, extended hospitalisation, and the need for critical care. Beyond its immediate impact, AL has also been associated with worse long-term oncological outcomes, particularly in individuals undergoing surgery for colorectal cancer [2].

Ensuring adequate bowel perfusion is fundamental for anastomotic healing, yet the traditional reliance on visual inspection and the surgeon’s judgment is subjective and may fail to identify impaired perfusion [3]. Indocyanine green fluorescence angiography (ICG-FA) has therefore gained attention as a novel intra-operative tool, providing real-time assessment of tissue vascularity through near-infrared imaging. By enabling surgeons to confirm well-perfused transection margins, ICG-FA has the potential to lower the risk of anastomotic failure.

Despite increasing use in clinical practice, the true magnitude of ICG-FA’s benefit remains uncertain. Several randomised controlled trials (RCTs) have evaluated its effectiveness in reducing AL, but many have been restricted by small sample sizes and methodological limitations, producing inconsistent outcomes [4]. In addition, anatomical and vascular differences between right-sided and left-sided resections create distinct baseline risks, raising the possibility that the benefit of ICG-FA may not be uniform across procedures.

This systematic review and meta-analysis of RCTs aims to determine whether ICG-FA reduces the risk of AL in colorectal surgery. A further objective was to explore whether its impact differs according to resection site, with the aim of clarifying the clinical value of this technology and identifying priorities for future research.

The findings of this systematic review and meta-analysis were initially presented as an oral presentation at the 6^th^ Edition of the Global Conference on Surgery and Anaesthesia (London, United Kingdom; May 16, 2025) and were subsequently selected for a distinction poster presentation at the Association of Laparoscopic Surgeons of Great Britain and Ireland (ALSGBI) Conference in November 2025).

## Review

Methods and materials

This systematic review and meta-analysis was conducted in line with the Preferred Reporting Items for Systematic Reviews and Meta-Analyses (PRISMA) 2020 [5] recommendations. No protocol was registered in the International Prospective Register of Systematic Reviews (PROSPERO) or other systematic review registries.

We included RCTs that compared intra-operative use of ICG-FA with conventional visual assessment during elective colorectal resections with primary anastomosis. Eligible studies were required to report AL as either a primary or secondary outcome.

The literature search was performed across five electronic databases: PubMed, Excerpta Medica database (Embase) via Ovid, Medical Literature Analysis and Retrieval System Online (MEDLINE) via Ovid, ScienceDirect, and ClinicalTrials.gov. Searches covered publications between July 1, 2015, and August 15, 2025. A combination of controlled vocabulary terms and free-text keywords was applied, including “indocyanine green,” “fluorescence angiography,” “colorectal surgery,” and “anastomotic leakage.” Only peer-reviewed full-text articles published in English were included. Non-randomised studies, conference abstracts, case reports, editorials, and review articles were excluded.

All retrieved records were imported into Rayyan software (Rayyan Systems Inc., Cambridge, MA) [6], a free web-based application designed for systematic reviews for screening and management. Duplicates were removed prior to the review process. Two authors (NW and ZAK) independently evaluated titles and abstracts, followed by full-text screening of potentially relevant studies. Any discrepancies were resolved in consultation with a third reviewer (KG).

Data extraction was carried out independently by two reviewers using a standardised form. Extracted information included study characteristics (author, year, country), patient demographics, surgical approach (laparoscopic, robotic, or open), type of colorectal resection, use of ICG-FA, incidence of AL, and duration of follow-up.

The methodological quality of included studies was assessed using the Cochrane Risk of Bias 2.0 (RoB 2) tool [7]. The tool is free and available under a Creative Commons Attribution-NonCommercial-No Derivatives 4.0 International License (CC BY-NC-ND 4.0).

Meta-analysis was performed with Review Manager (RevMan version 5.4, The Cochrane Collaboration, London, UK) [8], which is freely available for researchers. Odds ratios (OR) with 95% confidence intervals (CI) were calculated for dichotomous outcomes. Statistical heterogeneity was assessed using the I² statistic, with values above 50% considered indicative of substantial heterogeneity. Subgroup analyses were conducted to compare outcomes between left-sided and right-sided colorectal resections. The certainty of evidence for the primary outcome was appraised using the Grading of Recommendations Assessment, Development and Evaluation (GRADE) approach [9], which is freely available through the GRADE working group.

Results

From the initial search, 1,112 records were identified across the included databases (PubMed n=137, Embase n=692, MEDLINE Ovid n=174, ScienceDirect n=107, and ClinicalTrials.gov n=2). After removing 480 duplicates, 632 unique studies were screened using Rayyan software. Screening was performed independently by two reviewers (NW and ZAK) who assessed titles and abstracts before proceeding to full-text review. After screening, 533 articles were excluded.

**Figure 1:**
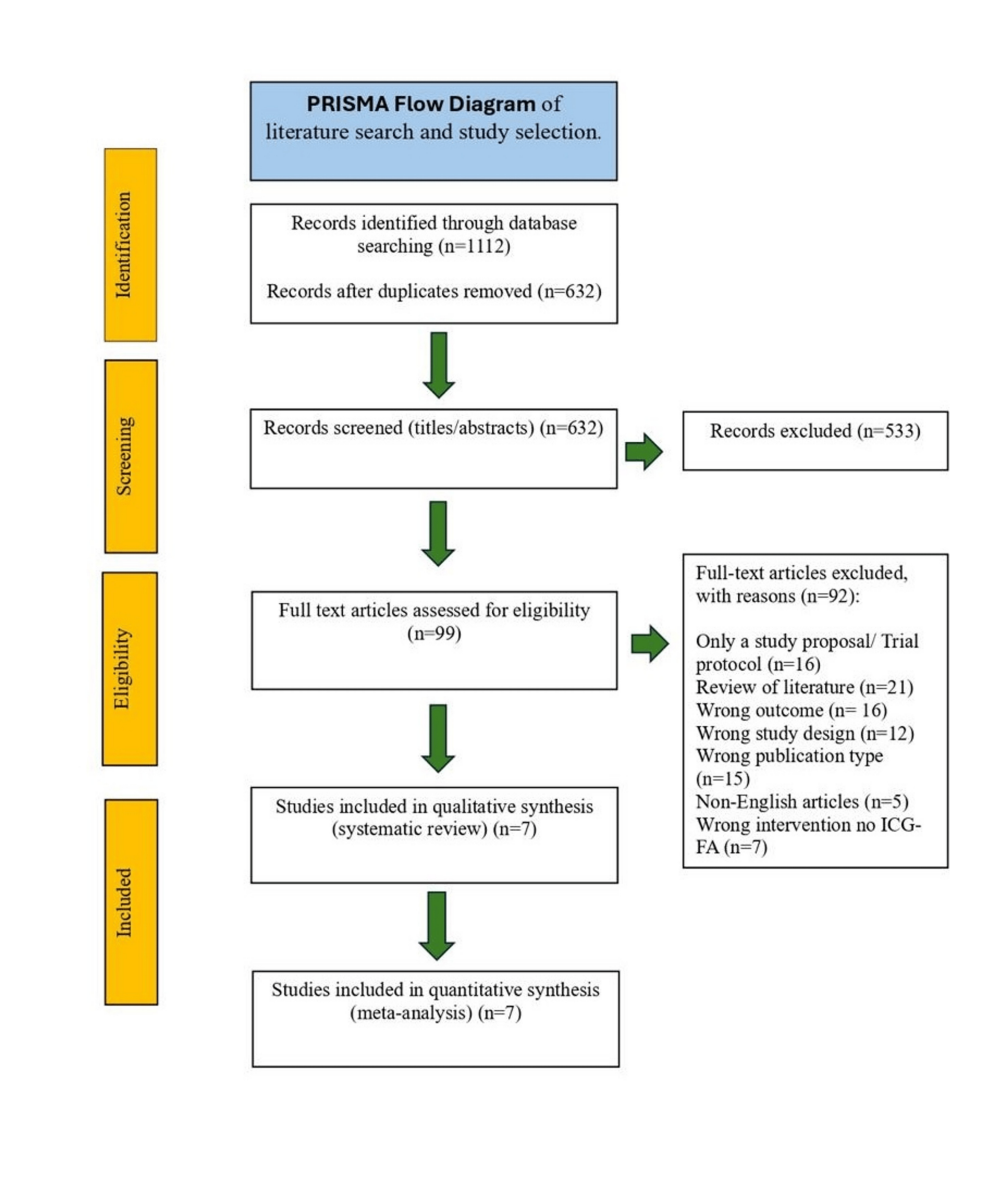
Study selection process based on PRISMA 2020 flow diagram PRISMA 2020 flow diagram illustrating the process of literature search, screening, eligibility assessment, and final study inclusion. Adapted from the PRISMA 2020 statement [5]. PRISMA: Preferred Reporting Items for Systematic Reviews and Meta-Analyses; n: number of records; RCT: randomised controlled trial.

A total of 99 full-text articles were assessed for eligibility, of which 92 were excluded. The reasons for exclusion were study protocols (n=16), review articles (n=21), outcomes unrelated to AL (n=16), unsuitable study design (n=12), publication type not meeting criteria (n=15), non-English articles (n=5), and studies without ICG-FA intervention (n=7). Ultimately, seven RCTs fulfilled the eligibility criteria and were included in the meta-analysis [10-16]. Any disagreements between reviewers were resolved through discussion. The overall selection process is depicted in the PRISMA 2020 flow diagram (Figure 1).

Seven RCTs were included [10-16], comprising 3,877 patients undergoing elective colorectal resection with primary anastomosis. Trials were conducted in Russia, Italy, the United States, the Netherlands, Finland, Japan, and Poland, with sample sizes ranging from 76 to 1,977 and enrolment spanning 2015-2023. Publication years ranged from 2020 to 2025. All studies compared ICG-FA with conventional visual perfusion assessment. Most procedures involved stapled anastomoses within 15 cm of the anal verge, with both benign and malignant cases represented. Surgical approaches were predominantly laparoscopic or robotic. AL, assessed 30-90 days postoperatively, was the primary outcome across studies. Key trial characteristics are summarised in Table 1.

**Table 1:** Characteristics of randomized controlled trials evaluating ICG-FA in colorectal surgery AL: anastomotic leak; ICG-FA: indocyanine green fluorescence angiography

Serial Number	Author	Year	Country	Trial Name	Period	Total Number of Participants	ICG-FA Group	Control Group	Population	Main Outcome (AL Rate)
1.	Alekseev et al. [11]	2020	Russia	FLAG	11.2017 to 08.2019	377	187	190	Patients undergoing elective stapled colorectal anastomosis within 15 cm of the anal verge for either benign or malignant conditions.	AL <30days; ICG:17, Control:31
2.	De Nardi et al. [13]	2020	Italy	-	01.2016 to 11.2017	240	118	122	Individuals receiving stapled colorectal anastomoses positioned 2-15cm from the anal verge, including both benign and malignant conditions.	AL <30days; ICG:6, Control:11
3.	Jafari et al. [12]	2021	USA	PILLAR III	03.2015 to 02.2017	347	178	169	Elective stapled anastomoses performed for malignant conditions at a distance up to 10cm from the anal verge.	AL <8 weeks; ICG:16, Control:16
4.	Faber et al. [15]	2024	Netherlands	AVOID Phase 3 Trial	07.2020 to 02.2023	931	463	468	Elective minimally invasive (laparoscopic or robotic) colorectal resections with planned primary anastomosis for malignant or benign colorectal conditions.	AL 90 days; ICG:32, Control:42
5.	Rinne et al. [16]	2025	Finland	ICG-COLORAL	09.2018 to 12.2023	1077	528	549	Elective laparoscopic colorectal resection with a primary anastomosis was included, while procedures involving the mid or lower rectum were excluded.	AL; ICG:31; Control:47
6.	Watanabe et al. [14]	2023	Japan	EssentiAL	12.2018 to 02.2021	839	422	417	Patients with clinically stage 0–III rectal carcinoma, less than 12 cm from the anal verge, scheduled for minimally invasive sphincter-preserving surgery	AL; ICG:32, Control:49
7.	Gach et al. [10]	2023	Poland	-	12.2020-08.2021	76	41	35	Rectal cancer with a distal tumour margin within 12 cm from the anal verge, treated by laparoscopic anterior resection with either partial or complete mesorectal resection.	AL; ICG:0, Control:3

Risk of bias was assessed using the Cochrane RoB 2.0 tool across seven RCTs. In one trial, Gach et al. [10] raised some concerns in multiple domains, particularly randomisation and selective reporting. Most trials (Alekseev et al. [11], Jafari et al. [12], De Nardi et al. [13], Watanabe et al. [14]) had some concerns, mainly due to allocation concealment. Two large trials (Faber et al. [15] and Rinne et al. [16]) were judged low risk. Across studies, the main issues were randomisation and selective reporting, while outcome measurement and missing data were generally low risk. The results of the risk of bias assessment are presented in Table 2.

**Table 2:** Risk of Bias (RoB) assessment of the included studies RoB was judged using the RoB 2 tool [7] at the study level for postoperative complications. Judgments were classified as low, some concerns, or high across five domains: D1 = randomisation process; D2 = deviations from intended interventions (effect of assignment); D3 = missing outcome data; D4 = outcome measurement; D5 = selection of the reported result. Overall risk of bias was rated low only if all domains were low; high if any domain was high; otherwise, some concerns.

Study	Randomization (D1)	Deviations (D2)	Missing Data (D3)	Outcome Measurement (D4)	Reporting (D5)	Overall RoB
Gach et al. [10] 2023 (WIITM)	Some concerns	Low	Some concerns.	Low	Some concerns	Some concerns
Alekseev et al. [11] 2020 (FLAG)	Some concerns:	Low	Low	Low	Some concerns	Some concerns
Faber et al. [15] 2024 (AVOID)	Low	Low	Low	Low	Low	Some concerns
Jafari et al. [12] 2021 (PILLAR III)	Some concerns	Low	Low	Low	Some concerns	Some concerns
De Nardi et al. [13] 2020	Some concerns	Low	Low	Low	Some concerns	Some concerns
Rinne et al. [16] 2025 (ICG- COLORAL)	Low	Low	Low	Low	Low	Low
Watanabe et al. [14] 2023 (EssentiAL)	Some concerns	Low	Low	Low	Low	Some concerns

**Figure 2:**
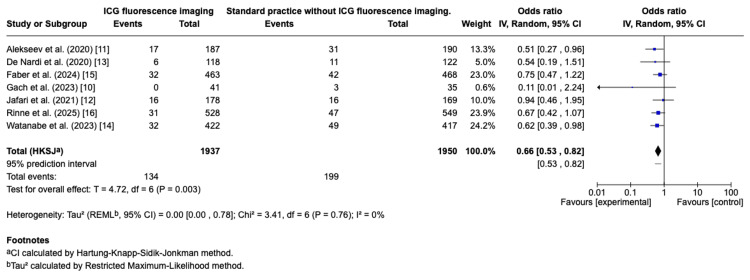
Overall effect of ICG-FA versus standard visual assessment on anastomotic leakage Forest plot displaying the combined results of trials evaluating indocyanine green fluorescence angiography (ICG-FA) against conventional visual assessment for anastomotic leak rates in colorectal surgery. Each square corresponds to the odds ratio (OR) of an individual study, with its area reflecting the relative study weight. Horizontal lines represent the 95% confidence interval (CI) for each trial. The diamond depicts the overall pooled effect, with its span indicating the 95% CI. Values of OR below 1 favour the use of ICG-FA, consistent with a reduction in anastomotic leakage. References: Alekseev et al. [11]; De Nardi et al. [13]; Jafari et al. [12]; Rinne et al. [16]; Watanabe et al. [14]; Gach et al. [10]; Faber et al. [15].

Meta-analysis of seven RCTs [10-16] including 3,887 patients showed a significantly lower rate of AL in the group undergoing ICG fluorescence-guided surgery compared with conventional assessment. The pooled OR was 0.66 (95% CI: 0.53 to 0.82; p = 0.003), indicating a 34% relative reduction in the odds of leak. Statistical heterogeneity was low (I² = 0%, p = 0.76), suggesting consistency of effect across studies. The 95% CI ranged from 0.53 to 0.82. Based on the absolute risk differences observed, the estimated number needed to treat (NNT) to prevent one anastomotic leak was 22. The summary of these results is presented in the forest plot (Figure 2).

**Figure 3:**
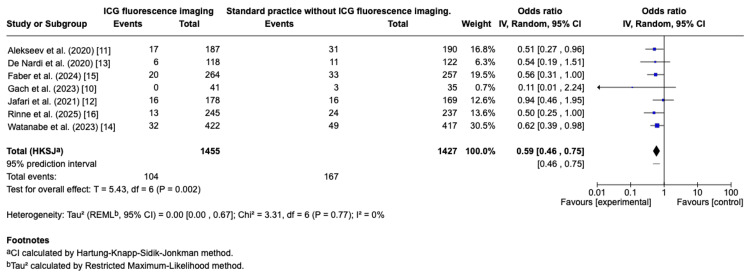
Effect of ICG-FA versus standard visual assessment on anastomotic leak rates in left-sided resections Forest plot summarising the comparison of indocyanine green fluorescence angiography (ICG-FA)  and conventional visual assessment for anastomotic leak rates in left-sided colorectal surgery. Each square corresponds to the odds ratio (OR) of a single trial, with its area scaled to represent the relative study weight, while horizontal lines mark the 95% confidence interval (CI). The diamond represents the combined OR with its CI, with values below 1 indicating a benefit of ICG-FA in lowering leak risk compared with standard assessment. References: Alekseev et al. [11]; De Nardi et al. [13]; Jafari et al. [12]; Rinne et al. [16]; Watanabe et al. [14]; Gach et al. [10]; Faber et al. [15]

For left-sided colorectal resections, ICG-FA was associated with a significant reduction in anastomotic leak rates (OR: 0.59, 95% CI: 0.46-0.75; p = 0.002), with no evidence of heterogeneity (I² = 0%). The NNT was calculated as 18. These findings are presented in the forest plot (Figure 3).

**Figure 4:**
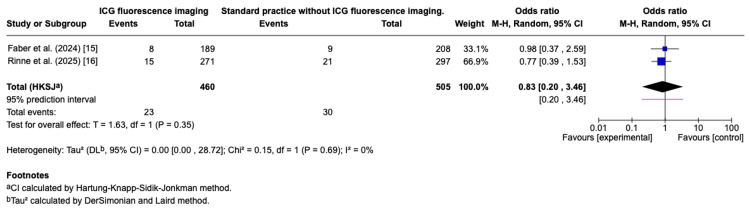
Effect of ICG-FA versus standard visual assessment on anastomotic leak rates in right-sided resections Forest plot presenting outcomes from trials that assessed indocyanine green fluorescence angiography (ICG-FA) versus visual inspection in rectal resections. Individual study odds ratios (ORs) are shown as squares, with the square size indicating the contribution of each trial to the overall analysis. Horizontal lines display the 95% confidence interval (CI) for each study. The diamond represents the pooled effect estimate with its CI, where OR values less than 1 reflect a trend toward reduced anastomotic leak rates with ICG-FA. References:  Faber et al. (2024) [15]; Rinne et al. (2025) [16].

For right-sided resections, no significant benefit was observed (OR: 0.83, 95% CI: 0.20-3.46; p = 0.35), with wide CIs indicating imprecision. Only 2 RCTs assessed the effect of ICG-FA on right-sided resections [15,16]. These findings may reflect lower baseline leak rates or smaller sample sizes in right-sided procedures. These findings were summarised in the forest plot (Figure 4).

Using the GRADE approach, the certainty of evidence for anastomotic leak was rated moderate. Most trials showed “some concerns” for risk of bias, mainly randomisation and selective reporting, leading to one downgrade. No serious concerns were noted for inconsistency, indirectness, and imprecision. Overall, evidence certainty was downgraded from high to moderate (Table 3).

**Table 3:** GRADE evidence profile for anastomotic leak Assessment of evidence quality for anastomotic leakage comparing indocyanine green fluorescence angiography (ICG-FA) with standard visual assessment in colorectal surgery. Seven randomised controlled trials were analysed. The Grading of Recommendations Assessment, Development and Evaluation (GRADE) framework was applied across the following domains: risk of bias, inconsistency, indirectness, imprecision, and publication bias. Overall certainty was judged as moderate, with some study-level bias but consistent findings and no clear publication bias.

Outcome	Number of Studies	Study Design	Risk of Bias	Inconsistency	Indirectness	Imprecision	Publication Bias	Certainty of Evidence
Anastomotic leak (ICG-guided vs. conventional surgery)	7	Randomised controlled trials	Serious (due to some concerns in many studies)	Not serious (effects are consistent across studies)	Not serious (studies directly address the question)	Not serious (narrow CIs, large sample size)	Undetected (no obvious small-study effects)	Moderate

Discussion

This meta-analysis of seven RCTs, including 3,887 patients, found that intra-operative use of ICG-FA significantly reduced AL compared with visual assessment. The pooled OR was 0.66 (95% CI: 0.53-0.82; p = 0.003), equating to a 34% relative reduction in odds of leakage. The absence of heterogeneity (I² = 0%) suggests consistency across diverse surgical settings. The estimated NNT was 22, underscoring tangible clinical benefit and supporting ICG-FA as a reliable adjunct to visual inspection.

Subgroup analysis demonstrated greater benefit in left-sided resections, with a 41% reduction in odds of leakage (OR: 0.59, 95% CI: 0.46-0.75; NNT = 18). No significant benefit was seen in right-sided resections (OR: 0.83, 95% CI: 0.20-3.46; p = 0.35), likely reflecting lower baseline risk and limited sample sizes.

The included trials spanned multiple countries and enrolled over 5,000 patients, representing diverse populations. Most involved stapled, minimally invasive anastomoses within 15 cm of the anal verge, aligning with modern surgical practice and enhancing generalisability.

Our findings align with recent high-quality evidence demonstrating that ICG-FA reduces AL in colorectal surgery. Elmajdub et al. [17] (2025) analysed five RCTs (n = 1,369) and confirmed significantly lower leakage rates with ICG-FA compared to visual assessment. Similarly, Lucarini et al. [18] (2024) reviewed four RCTs (n = 1,510) in rectal cancer and reported leakage rates of 9.0% with ICG-FA versus 13.9% in controls (risk ratio (RR) ≈ 0.5, p = 0.003), representing a 4.5% absolute reduction. Broader reviews by McEntee et al. [19] (2025) and Hussain et al. [20] (2025) also support reduced leak rates. Our analysis adds clinical value, estimating NNTs of 22 overall and 18 for left-sided resections.

Several recent studies have further highlighted the clinical value of ICG fluorescence imaging in reducing the risk of anastomotic complications. Chen et al. [21] demonstrated in a large propensity score-matched cohort that ICG angiography lowered the incidence of AL following trans-anal total mesorectal excision. Similarly, Maione et al. [22] emphasised the evolving role of near-infrared fluorescence in colorectal surgery, noting that it is increasingly recognised as a valuable adjunct to conventional surgical assessment. In the context of minimally invasive approaches, Su et al. [23] reported that ICG fluorescence reliably assessed bowel perfusion during laparoscopic colon cancer surgery, while Shapera et al. [24] confirmed its feasibility and utility in robotic-assisted colorectal procedures. Furthermore, Meyer et al. [25] reviewed current practices and underscored the importance of ICG in addressing long-standing challenges in anastomotic leak prevention. Finally, the GRADE Working Group’s consensus [26] on evaluating evidence quality reinforces the necessity of integrating robust, standardised methods when assessing new technologies such as ICG fluorescence. Collectively, these findings strengthen the evidence base supporting ICG as an adjunctive tool to enhance intra-operative decision-making and potentially improve anastomotic outcomes.

Unlike earlier reviews that often combined randomised and non-randomised data, this study synthesises evidence exclusively from RCTs, providing a stronger level of reliability. Limiting the analysis to RCTs reduces bias from retrospective designs and produces more robust estimates. In addition, our review contributes new clinical context by calculating the NNT and by comparing outcomes between left- and right-sided resections. Together, these insights refine the current evidence base and suggest that the protective effect of ICG-FA is most relevant in left-sided colorectal procedures, whereas further large-scale RCTs are still required to clarify its role in right-sided surgery.

This review highlights the practical value of ICG-FA in colorectal surgery. By providing real-time, objective perfusion assessment, ICG-FA reduces reliance on subjective visual judgement and lowers the risk of AL, particularly in left-sided resections where baseline risk is highest. Its adoption could improve patient safety, decrease re-intervention rates, and shorten recovery, thereby reducing overall healthcare costs. Integration of ICG-FA into surgical pathways, including Enhanced Recovery After Surgery (ERAS) programmes, may further optimise outcomes. Future research should standardise protocols, evaluate fluorescence thresholds, and assess cost-effectiveness to support its broader incorporation into routine colorectal practice.

Strengths and Limitations

This review provides the most up-to-date synthesis of randomised evidence (to August 2025) on the role of ICG-FA in preventing AL in colorectal surgery. Unlike many previous reviews, it exclusively included RCTs, thereby enhancing methodological rigour and reducing the risk of bias inherent to observational studies. The review adhered to PRISMA 2020 guidelines and applied robust methodology. A large sample size (n = 3,887) across multiple international centres improves external validity. Subgroup analyses provided clinically relevant insights, demonstrating that ICG-FA is most beneficial in left-sided resections. Low statistical heterogeneity (I² = 0%) strengthens the robustness of findings.

This review has several important limitations. Variability in ICG administration protocols, fluorescence interpretation, and imaging systems across trials may have influenced the reported outcomes. Definitions of AL and follow-up durations, ranging from 30 to 90 days, were not fully standardised, which could introduce measurement bias. Subgroup analyses, particularly for right-sided resections, were based on relatively small sample sizes, limiting statistical power to detect differences. Finally, this review was not prospectively registered in PROSPERO; however, adherence to PRISMA 2020 guidelines ensured methodological transparency and strengthened the validity of the findings.

## Conclusions

This systematic review and meta-analysis provides evidence that ICG-FA significantly lowers the risk of AL in colorectal surgery, with the strongest benefit observed in left-sided resections. These results highlight the value of ICG-FA as a complementary tool to conventional visual assessment, supporting intraoperative decision-making and potentially enhancing surgical safety.

AL is considered one of the most serious postoperative complications in colorectal surgery, contributing to higher morbidity, mortality, reoperation rates, and healthcare costs. The reduction in leakage rates associated with ICG-FA suggests an important step forward in improving both clinical outcomes and resource utilisation.

Nevertheless, variability in protocols, dosing, and assessment methods across studies introduces heterogeneity, and the evidence for right-sided resections remains less consistent. Large, multicentre randomised trials are needed to standardise techniques, provide objective quantification, and evaluate cost-effectiveness as well as long-term outcomes.

In summary, ICG-FA appears to be a safe and effective adjunct that reduces the risk of AL and holds promise for improving the overall quality of colorectal surgical care.
